# Protein Domain-Level Landscape of Cancer-Type-Specific Somatic Mutations

**DOI:** 10.1371/journal.pcbi.1004147

**Published:** 2015-03-20

**Authors:** Fan Yang, Evangelia Petsalaki, Thomas Rolland, David E. Hill, Marc Vidal, Frederick P. Roth

**Affiliations:** 1 Department of Molecular Genetics, University of Toronto, Toronto, Ontario, Canada; 2 Donnelly Centre, University of Toronto, Toronto, Ontario, Canada; 3 Lunenfeld-Tanenbaum Research Institute, Mt. Sinai Hospital, Toronto, Ontario, Canada; 4 Center for Cancer Systems Biology (CCSB), Dana-Farber Cancer Institute, Boston, Massachusetts, United States of America; 5 Department of Genetics, Harvard Medical School, Boston, Massachusetts, United States of America; 6 Canadian Institute for Advanced Research, Toronto, Ontario, Canada; 7 Department of Computer Science, University of Toronto, Toronto, Ontario, Canada; Princeton University, United States of America

## Abstract

Identifying driver mutations and their functional consequences is critical to our understanding of cancer. Towards this goal, and because domains are the functional units of a protein, we explored the protein domain-level landscape of cancer-type-specific somatic mutations. Specifically, we systematically examined tumor genomes from 21 cancer types to identify domains with high mutational density in specific tissues, the positions of mutational hotspots within these domains, and the functional and structural context where possible. While hotspots corresponding to specific gain-of-function mutations are expected for oncoproteins, we found that tumor suppressor proteins also exhibit strong biases toward being mutated in particular domains. Within domains, however, we observed the expected patterns of mutation, with recurrently mutated positions for oncogenes and evenly distributed mutations for tumor suppressors. For example, we identified both known and new endometrial cancer hotspots in the tyrosine kinase domain of the FGFR2 protein, one of which is also a hotspot in breast cancer, and found new two hotspots in the Immunoglobulin I-set domain in colon cancer. Thus, to prioritize cancer mutations for further functional studies aimed at more precise cancer treatments, we have systematically correlated mutations and cancer types at the protein domain level.

## Author Summary

Extensive tumor genome sequencing has provided raw material to understand mutational processes and identify cancer-associated somatic variants. However, fundamental problems remain to: i) separate ‘driver’ from ‘passenger’ mutations, ii) further understand the functional mechanisms and consequences of driver mutations, and iii) identify the cancer types in which each driver mutation is relevant. Here we analyze whole-genome and exome tumor sequencing data from the perspective of protein domains—the basic structural and functional units of proteins. Exploring the cancer-type-specific landscape of domain mutations across 21 cancer types, we identify both cancer-type-specific mutated domains and mutational hotspots. Frequently-mutated domains were identified for oncoproteins for which the ‘mutational hotspot’ phenomenon owing to the relative rarity of gain-of-function mutations is well known, and also for tumor suppressor proteins, for which more uniformly distributed loss-of-function driver mutations are expected. A given gene product may be perturbed differently in different cancers. Indeed, we observed systematic shifts between cancer types of the positions at which mutations occur within a given protein. Both known and novel candidate driver mutations were retrieved. Novel cancer gene candidates significantly overlapped with orthogonal systematic cancer screen hits, supporting the power of this approach to identify cancer genes.

## Introduction

Cancer is caused in large part by the accumulation of mutations in oncogenes and tumor suppressor genes. Previous analyses of well-studied cancers, such as colorectal cancer and retinoblastoma, have suggested that as few as three mutations are sufficient for cancer initiation [1–3]. Thousands of cancer genomes have now been sequenced[4, 5], including efforts from The Cancer Genome Atlas (TCGA) and the International Cancer Genome Consortium (ICGC) [6, 7]. In recent years, the genetic landscape of mutations has been revealed in several well-studied cancers [8–16]. However, the process of extracting useful knowledge from this vast sequence resource has only begun.

The complexity of cancer genomes represents a challenge to therapy and our basic understanding of the disease and therefore also to therapy. Individual cancers can contain thousands of somatic mutations [17, 18], only a small fraction of which are likely to be driver mutations contributing to tumor initiation or progression [18–22]. Even genes that are well known to cause cancer contain many effectively-neutral passenger mutations [23]. For example, it was reported that 80% of non-synonymous single-base substitutions observed in genes encoding protein kinases are passenger mutations [24]. Most candidate driver gene identification has been done on the basis of observing mutations in a large fraction of tumor samples. However, the list of putative driver mutations generated includes many that are implausible, with a false positive rate that increases with the number of sequenced tumor samples [25, 26].

Determining the effect of mutations on the structure and function of proteins remains challenging [27]. Previous gene-based studies have generally focused on the whole gene or whole protein, but mutations in different protein domains, structural units that often have distinct functions, may have different functional consequences. Thus, gene-level analysis can identify genes that contribute to multiple cancers, but does not map mutations to structural elements. Recently, computational structural studies have explored mutational effects on specific regions of a protein (e.g., the binding site)[28–31]. For example, Joerger and Fersht showed that certain mutations in the p53 protein can determine folding state and affinity of p53 for specific target DNA elements. Also, different p53 mutations affect different protein–protein interaction interfaces dictating either tetramerization of p53 or its interaction with a multitude of other regulatory proteins[30]. Similarly, the effects of mutations in different protein kinase sub-domains have been shown to have different functional impacts[28]. Thus, within a multi-functional gene, different mutations can affect different functions. The structural details of individual mutants can provide the basis for the design of cancer therapeutics[30]. Indeed, a given gene may have different functional roles in different cancers, reflected in shifts in the mutational distribution of different cancers. Recently, Nehrt *et al*. examined 100 colon cancer and 522 breast cancer samples to identify specific domain types with heightened mutation rates, succeeding even within genes that have generally lower mutation rates in colon or breast cancer[32, 33]. Mutations occurring within a particular domain are more likely to share structural and functional effects [15]. Two mutations within a given gene may be associated with different human diseases, e.g., potentially by disrupting different protein interactions [34, 35]. Thus, studies that consider mutational positions (e.g., relative to known domains) could be beneficial in elucidating functional effects of mutations.

In this study, a “domain instance” refers to a particular protein domain encoded within a particular gene and a “domain type” refers to a Pfam domain ‘pattern’ that may correspond to different domain instances encoded by different genes. In other words, a domain instance refers to a specific amino acid subsequence within a given single protein that matches to a given domain type.

To better distinguish this study from previous related studies, such as the domain landscape in colon and breast cancer by Nehrt *et al*, we note that we are systematically analyzing multiple (twenty-one) cancer types. Like Nehrt *et al*., we analyze each Pfam domain type. In addition, however, we specifically analyze each Pfam domain instance. Rather than simply seeking domains with a mutational density that is enriched relative to other genomic regions, we further require that this enrichment is greater in one cancer type than in all other cancer types. This has the advantage of pointing us to interesting differences between cancer types, while also implicitly controlling for region-specific differences in background mutation rate. Thus, we are analyzing the ‘domain-centric mutational landscape’ by examining the domain-level distribution of missense somatic mutations across multiple cancer types.

**Fig 1:**
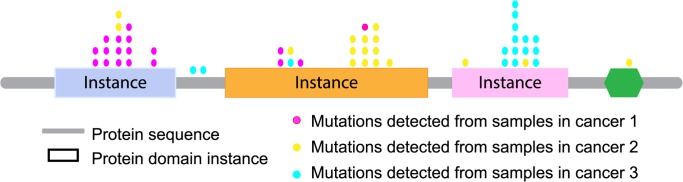
Mapping mutations detected from different cancers to domain instances. Rectangles represent protein domain instances in a given gene. Colored dots represent mutations detected in different cancer types.

We mapped missense somatic mutations to domain instances (Fig. 1. outlines this process) for 21 cancer types (Table 1) and detected 100 cancer-type-specific significantly-mutated domain instances (SMDs) among different cancers. Further examination of these 100 domain instances showed that the proportion of within-domain mutations corresponding to hotspot positions can distinguish oncoproteins from tumor suppressor proteins. We also found that the vast majority of within-domain mutational hotspots shared by multiple cancer types occurred at functional sites. Thus, domain mutational landscape information can be used to prioritize candidate cancer-causing mutations and to elucidate their cancer-type-dependent functional effects.

**Table 1:** Prevalence of predicted damaging mutations in domain instances among cancer types.

Cancer Type	Mutation Counts	Gene Counts	Domain Families
Neuroblastoma	**166**	**154**	**132**
Chondrosarcoma	**68**	**49**	**47**
Breast cancer	**4026**	**2568**	**1293**
Glioblastoma and medulloblastoma	**1423**	**911**	**559**
Cervical cancer	**707**	**570**	**410**
Endometrial carcinoma	**12183**	**6105**	**2451**
Lymphoma and leukemia	**1443**	**692**	**468**
Renal cell carcinoma.	**4774**	**3241**	**1591**
Colorectal cancer	**27132**	**9706**	**3426**
Liver cancer	**485**	**309**	**235**
Lung cancer	**11654**	**5899**	**2401**
Meningioma	**89**	**66**	**62**
Esophageal adenocarcinoma	**935**	**568**	**366**
Ovarian cancer	**4182**	**3035**	**1395**
Pancreatic cancer	**703**	**534**	**362**
Prostate cancer	**1917**	**1409**	**785**
Adenoid cystic carcinomas	**165**	**126**	**107**
Melanoma	**1824**	**1352**	**758**
Striated muscle	**16**	**13**	**12**
Head and neck squamous cell carcinoma	**582**	**445**	**307**
Bladder cancer	**2996**	**1289**	**804**

## Results

The results of this study fall into four areas: 1) exploration of cancer-type-specific domain mutation landscapes across 21 cancer types; 2) identification of cancer-type-specific shifts in mutation position within given proteins; 3) comparison of domain-centric mutational patterns between oncoproteins and tumor suppressor proteins; and 4) correlation of mutational hotspots occurring in multiple cancer types to oncogenicity and functional roles.

### Cancer-type-specific domain mutation landscapes across 21 cancer types

Protein domains are generally regarded as the conserved structural and functional units of proteins. We therefore focused on the 237,716 missense somatic mutations, across 21 different human tissues, that fell within protein domain instances. We further focused on the subset of 76,158 mutations that were predicted to compromise the function of the harboring protein, using the IntOGen–mutation platform[36] (Table 1, S1 Table). To avoid observational biases, the above-mentioned mutations were derived only from genome-scale (either whole-genome or exome) sequencing studies (listed in Table 2).

**Table 2:** Patient tissue samples from selected cancer genome studies across 21 cancer types.

Primary Site	Cancer Type	Sample Counts	References
Autonomic ganglia	**Neuroblastoma**	**134**	**[89, 98]**
Bone	**Chondrosarcoma**	**66**	**[85]**
Breast	**Breast cancer**	**978**	**[70, 86, 96, 117]**
Central nervous system	**Glioblastoma and medulloblastoma**	**525**	**[81, 91, 93, 103, 106, 125, 126]**
Cervix	**Cervical cancer**	**14**	**[92]**
Endometrium	**Endometrial carcinoma**	**261**	**[104, 123]**
Hematopoietic and lymph	**Lymphoma and leukemia**	**415**	**[80, 82, 97, 103, 115, 121]**
Kidney	**Renal cell carcinoma.**	**594**	**[104, 113, 116, 119, 120]**
Colon	**Colorectal cancer**	**762**	**[71, 79, 87, 129]**
Liver	**Liver cancer**	**531**	**[84, 101, 111, 114]**
Lung	**Lung cancer**	**825**	**[72, 73, 88, 90, 99, 130]**
Meninges	**Meningioma**	**39**	**[122]**
Esophagus	**Esophageal adenocarcinoma**	**242**	**[120, 131]**
Ovary	**Ovarian cancer**	**637**	**[107, 125]**
Pancreas	**Pancreatic cancer**	**202**	**[83, 109]**
Prostate	**Prostate cancer**	**423**	**[8, 100, 127, 130]**
Salivary gland	**Adenoid cystic carcinomas**	**60**	**[112]**
Skin	**Melanoma**	**133**	**[14, 105]**
Soft tissue	**Striated muscle**	**13**	**[103]**
Upper aero-digestive tract	**Head and neck squamous cell carcinoma**	**203**	**[118, 132]**
Urinary tract	**Bladder cancer**	**203**	**[110]**

**Fig 2:**
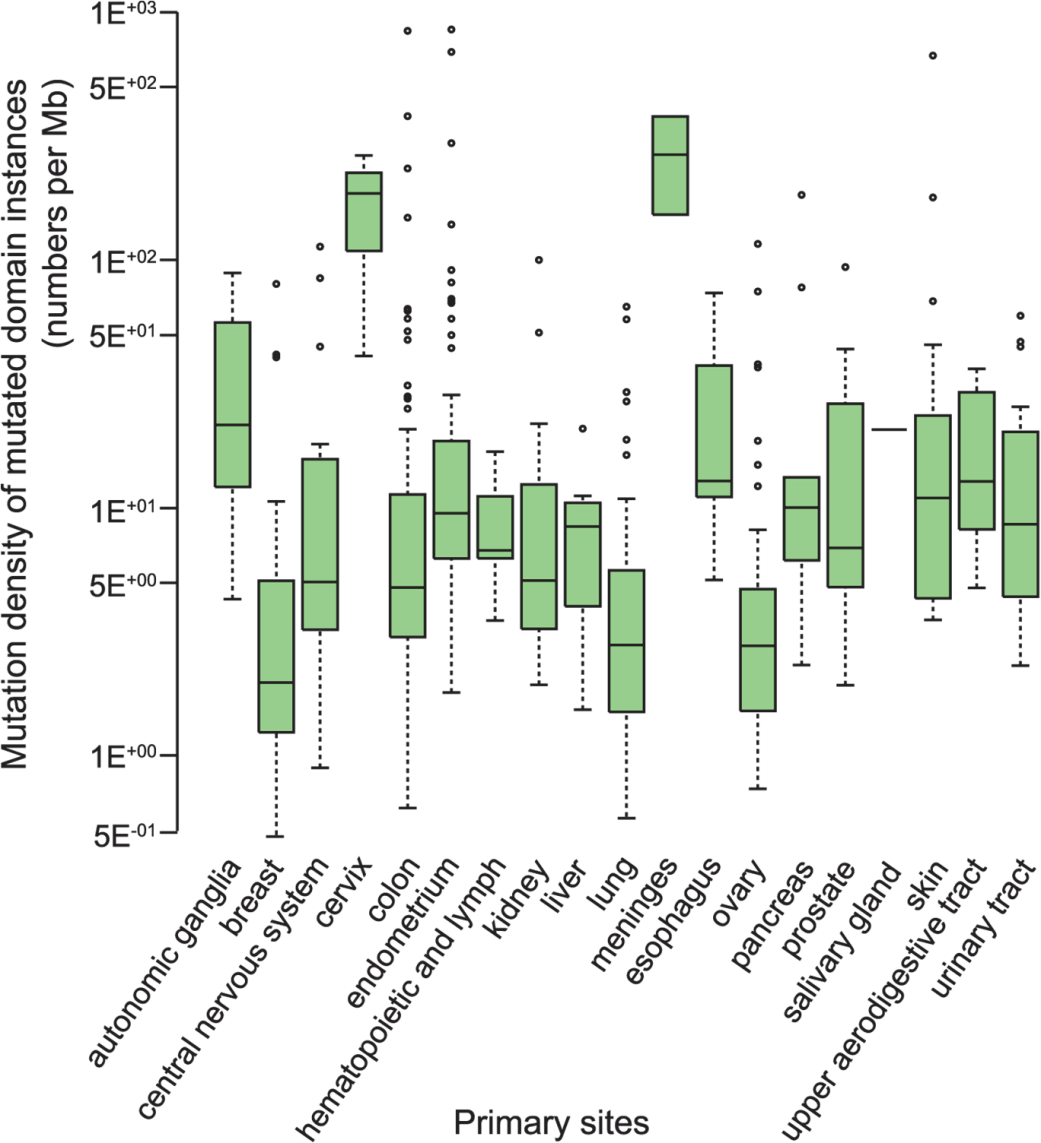
Mutation Densities for Domain Instances across Cancers. Box plots display mutation densities for mutated domain instances in different cancers. Outliers are shown as dots. Only predicted- damaging mutations predicted by IntOGen were used for this analysis (S1 Table).

The prevalence of missense somatic mutations can vary from cancer to cancer at the domain level [37, 38]. We found that most domain instances had a mutational density of only one or two missense somatic mutations per megabase in the corresponding DNA sequence (Fig. 2.). For example, a mutated domain of length 209 residues (the average domain instance length) contains an average of one single-amino acid-changing mutation for every 71 patients. Although domains with high mutation rates can be seen for many cancers (Fig. 2.), these mutation rates can be misleading. Given heterogeneity of mutation rates across the genome and differences in overall mutation rate for different cancers, domain-instances with the highest mutation density in a given cancer may not be the true drivers of cancer progression[25].

To control for both positional and cancer-type specific differences in mutation rate, we sought domain instances that were highly mutated relative both to the same domain instance in other cancer types and also to other domain instances within the same cancer type (see Materials and Methods). We identified ∼100 cancer-type-specific significantly mutated domain instances (SMDs) in 21 cancer types (S2 Table; *P*-value = 10^–7^, Fisher’s Exact test, False Discovery Rate (FDR) <0.05). The number of cancer-type-specific SMDs in each of the 21 cancer types is listed in Table 3, and in S3 Table in greater detail. With only two exceptions, the smallest number of mutations observed for a domain instance that was declared to be significantly mutated was 6. The exceptions were the Collagen domain instance (with only 2 mutations) within the *COLEC11* gene product in soft tissue cancers, for which only 14 samples were available; and the CCDC14 domain instances (with 3 mutations) encoded by *CCDC14* in cancers of the salivary gland, for which only 60 samples were available (S4 Table).

**Table 3:** Significantly mutated domain instances and corresponding genes in each cancer type.

Cancer Type	Significantly Mutated Domain Instance Counts	Related Gene Counts
Neuroblastoma	1	1
Chondrosarcoma	5	5
Breast cancer	4	4
Glioblastoma and medulloblastoma	11	9
Cervical cancer	4	4
Colorectal cancer	3	3
Endometrial carcinoma	11	10
Lymphoma and leukemia	27	26
Renal cell carcinoma.	5	5
Liver cancer	3	2
Lung cancer	9	9
Meningioma	3	3
Esophageal adenocarcinoma	4	4
Ovarian cancer	1	1
Pancreatic cancer	1	1
Prostate cancer	8	8
Adenoid cystic carcinomas	1	1
Melanoma	5	5
Striated muscle	1	1
Head and neck squamous cell carcinoma	3	3
Bladder cancer	1	1

We found between 3 and 7 SMDs for each cancer type, except for endometrial cancer (with 11 SMDs) as well as hematopoietic and lymphatic cancer (**with 27** SMDs). Of the 94 genes encoding at least one SMD, 40 (42%) had already been implicated in cancer according to the Sanger Cancer Gene Census (‘Cancer Census’) [39, 40], including well-established cancer-causing genes such as *KRAS, EGFR* and *TP53*. Enrichment for Cancer Census genes was both strong and significant (∼12-fold enrichment; *P*-value = 5×10^–34^, Fisher’s Exact test), and suggests the remaining 54 genes that are not already known to be cancer drivers represent good candidates. For example, the Syntaphilin protein, encoded by *SNPH* harbors the syntaphilin domain instance, which was significantly mutated in lung cancer. Despite reports that it is brain-specific[41], *SNPH* is expressed in lung according to microarray[42, 43] and RNA-seq studies[44].

We compared the resulting novel cancer gene candidates with cancer gene candidates emerging from a large-scale *in vivo* (mouse) screen via mutagenesis with Sleeping Beauty transposons [45]. Of the 94 genes encoding cancer type-specific SMDs, 24 were found in the Sleeping Beauty dataset (∼3-fold enrichment; *P*-value = 7×10^–06^, Fisher’s Exact test). Of the subset of 54 candidate genes not already known to be cancer genes, 10 were found in the Sleeping Beauty dataset (∼2-fold enrichment; *P*-value = 5×10^–3^, Fisher’s Exact test, Table 4).

**Table 4:** Genes that encode cancer-type-specific significantly mutated domain instance and overlap with the Sleeping Beauty dataset.

Gene Symbol	SignificantlyMutated Domain		Cancer Type	Predicted Category	Reported Category
***CNTN4***	PF13895.1	Lymphoma and leukemia		Tumor suppressor	Not determined
***CTNNA3***	PF01044.14	Lymphoma and leukemia		Tumor suppressor	Not determined
***EPHA6***	PF14575.1	Lymphoma and leukemia		Tumor suppressor	Not determined
***FOXO1***	PF00250.13	Lymphoma and leukemia		Tumor suppressor	Tumor suppressor
***GNA13***	PF00503.15	Lymphoma and leukemia		Oncogene	Not determined
***MAGI1***	PF00503.15	Lymphoma and leukemia		Tumor suppressor	Tumor suppressor
***PCDH11X***	PF08266.7	Lymphoma and leukemia		Oncogene	Not determined
***PCDH11X***	PF00028.12	Glioblastoma, medulloblastoma		Tumor suppressor	Not determined
***PTPRD***	PF00102.22	Prostate cancer		Tumor suppressor	Not determined
***SMAD4***	PF03166.9	Colorectal cancer		Tumor suppressor	Tumor suppressor
***SMAD4***	PF03166.9	Esophageal adenocarcinoma		Tumor suppressor	Tumor suppressor
***USP25***	PF00443.24	Liver cancer		Oncogene	Not determined

**Fig 3:**
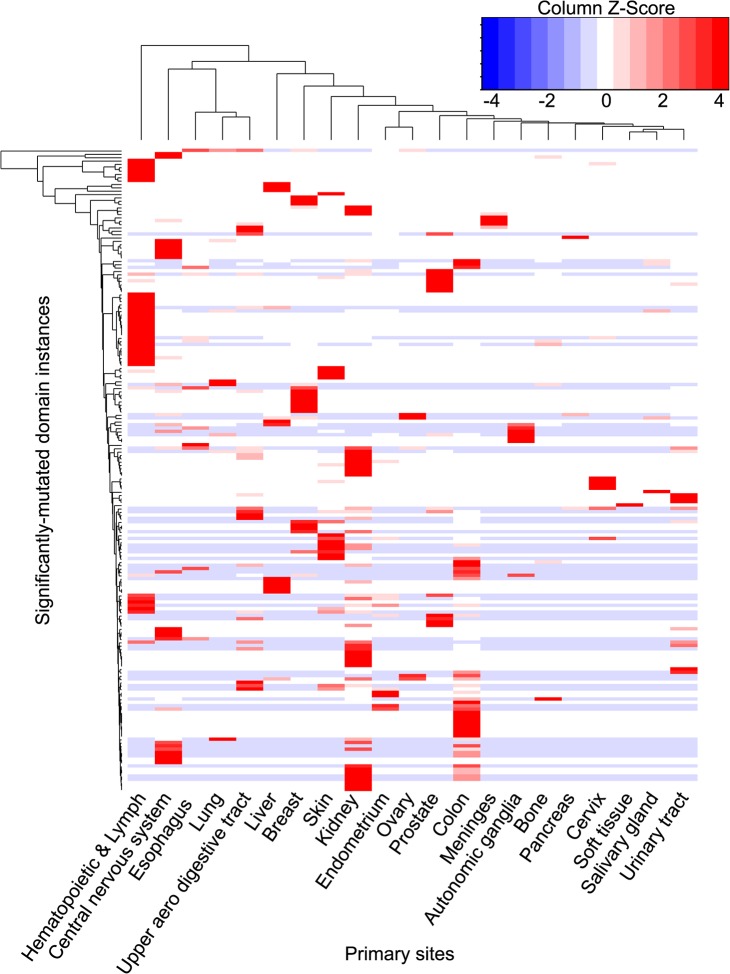
Clustering of significantly-mutated domain instances across 21 cancer types. The heatmap reflects the significance of cancer-type-specific mutation density of each domain instance in different cancers. Side bars in the same color indicate domain instances encoded by the same gene, and domain instances belonging to the same domain type.

The distribution of cancer-type-specific SMDs varies across cancer types. Among cancer-type-specific SMDs, most (95%) were only significantly mutated in a single cancer type (Fig. 3.). Five domain instances were found to be significantly mutated in more than one cancer (Table 4): a Ras domain instance of *KRAS*, mutated in lung and pancreatic cancer; a PHD finger domain instance (zf-HC5HC2H) of MLL3, mutated in breast and prostate cancer; a MAD homology 2 (MH2) domain instance of *SMAD4*, mutated in colon and esophageal cancer; a SNF2 family N-terminal (SNF2_N) domain instance of *SMARCA4*, mutated in esophageal cancer and cancer of the central nervous system; and the P53 DNA binding domain instance of *TP53*, mutated in 8 cancer types. With the exception of *KRAS*, these genes are usually regarded as tumor suppressors. This suggests that, while tumor suppressors may cause different cancers via a common loss of function mechanism, the gain-of-function mechanism of oncogenes is more likely to be tissue-specific.

### Cancer-type-specific positioning of mutations within a given gene

Domain instances mutated in a specific cancer type can point to functions that are specifically disrupted in that cancer type. Furthermore, the observation that a given gene product has different domain instances mutated in different cancer types may elucidate how a single gene can play different roles in different cancers. To identify candidate genes with this behavior, we first selected all of the multi-domain genes that contained at least one cancer-type-specific SMD, and examined the cancer-type-specificity of these domains (see Materials and Methods).

Among the 94 genes identified above to contain cancer-type-specific SMDs, 52 genes had multiple domain instances with differing cancer-type-specificity (see S2 Table). These 52 genes were enriched for evidence of involvement in cancer, with 16 being Cancer Census genes (enrichment factor ∼ 11.9; *P*-value = 6.7 ×10^–13^, Fisher’s Exact test), and 15 being candidate cancer genes according to the Sleeping Beauty screen (enrichment factor ∼ 4.5; *P*-value = 1.9 ×10^–6^, Fisher’s Exact test).

**Fig 4:**
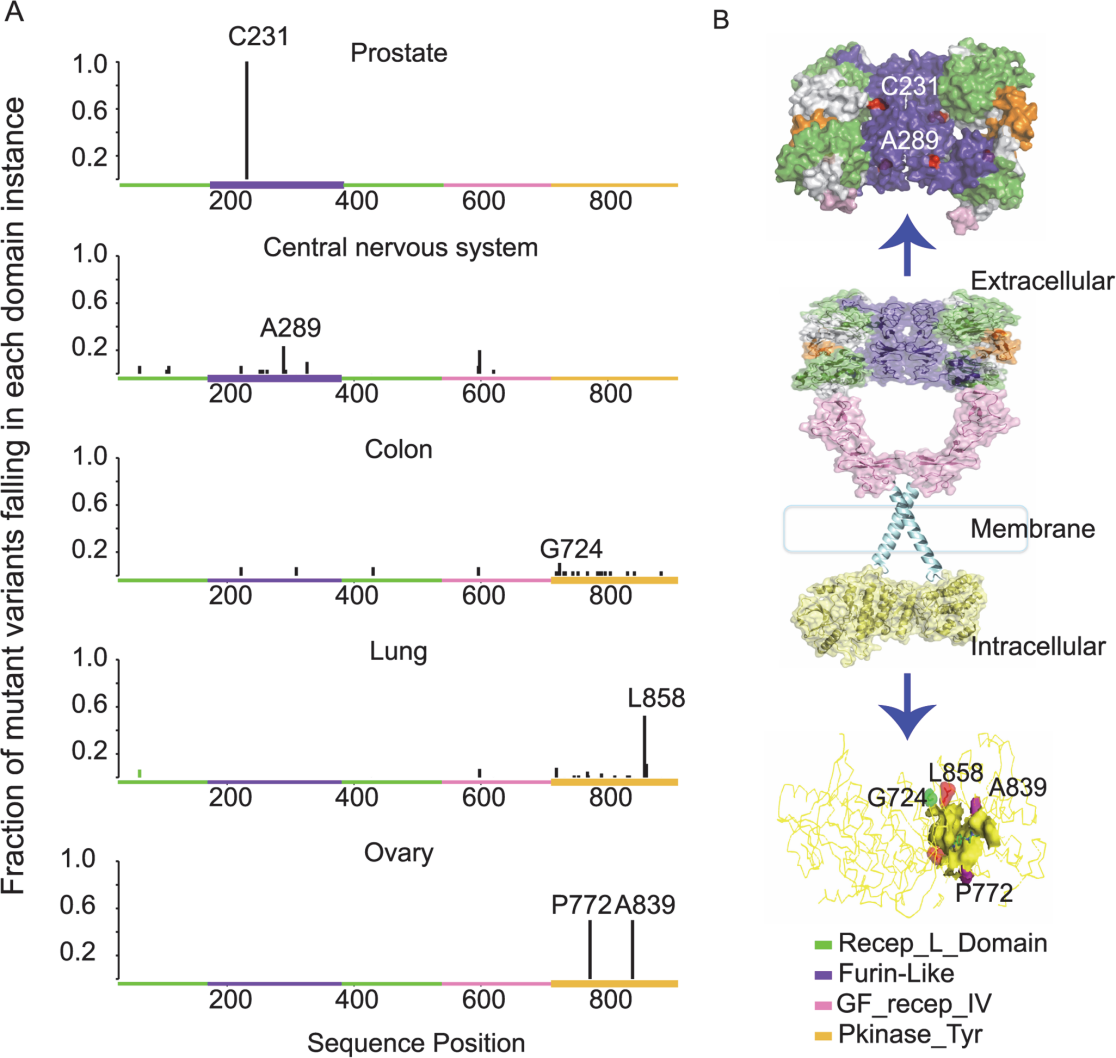
Mutations in EGFR across 5 different cancers with protein structure context. (A) The histogram displays the proportions of mutation counts detected at each residue to the total number of mutations that fall in the four different domains encoded by the gene EGFR, in five different cancers. The x-axis indicates the position of mutant residues. Mutations in different domains are shown in different colors. (B) shows the structure of the EGFR protein with epidermal growth factors colored in orange. The arrows point to enlargments of portions of the protein. The tails of the kinase domain are not shown in this structure. The structure visualization was based on Protein Data Bank structure models 1nql, 1ivo, 2jwa, 1m17 and 2gs6[47–50]. Significantly-mutated domain instances (SMDs) were shown as thicker boxes.

To illustrate this analysis, we show the distribution of domain mutations in the EGF receptor, encoded by *EGFR*, across five cancers (Fig. 4A.). The EGF receptor is a flexible protein with four distinct domains, including extracellular and transmembrane regions, the intracellular kinase domains, and a long flexible tail (Fig. 4B.). Our analysis recapitulated domain mutation patterns seen in corresponding to previous findings. The extracellular region consists of the furin-like (Furin-Like) domain, the growth factor receptor domain IV (GF_recep_IV) and the L domain (Recep_L_Domain). The Furin-Like and the GF_recep_IV domains were both found to be significantly mutated in cancers of the central nervous system. Mutations in the extracellular region of EGF receptor have been associated with ligand-independent dimerization in cancers of the central nervous system[46], and mutations in the intracellular region of EGF receptor are associated with sensitivity to kinase inhibitors[46].

In lung cancers, mutations were significantly enriched in the tyrosine kinase domain (Pkinase_Tyr). This is a well-known location for oncogenic mutations that hyper-activate downstream pro-survival signaling pathways in lung cancer by causing the auto-phosphorylation of C-terminal residues [51].

The detailed functional consequences of *EGFR* mutations in prostate and colon cancer are still unclear. Differences in the positions of mutations between the extracellular (glioblastoma and prostate cancer) and intracellular regions (colon, lung and ovarian cancer) of the EGF receptor in different cancer types suggest different oncogenic mechanisms and possibly different therapeutic avenues.

Other interesting examples included that of the histone-lysine N-methyltransferase MLL3 protein, for which the PHD finger domain is mutated in breast cancer and prostate cancer, and for which the SET domain is mutated in glioblastoma and medulloblastoma. MLL3 is reported to possess histone methylation activity and is also involved in transcriptional co-activation. Knockdown or deletion of *MLL3* using RNAi or CRISPR is reported to cause acute myeloid leukemia in a mouse model [52].

Domain-associated mutational biases have been reported in several studies focusing on single well-known cancer genes such as the *PI3KCA* gene in colon and breast cancer[32], and the *NOTCH1* gene in leukemia, breast and ovarian cancer [53]. Here, we analyzed the distribution of somatic missense mutations for 14,083 genes across 21 cancer types and identified 52 genes (36 of which are not yet known to be cancer genes) for which different domain instances may contribute to different cancer types.

### Mutational trends of oncoproteins and tumor suppressor proteins

**Fig 5:**
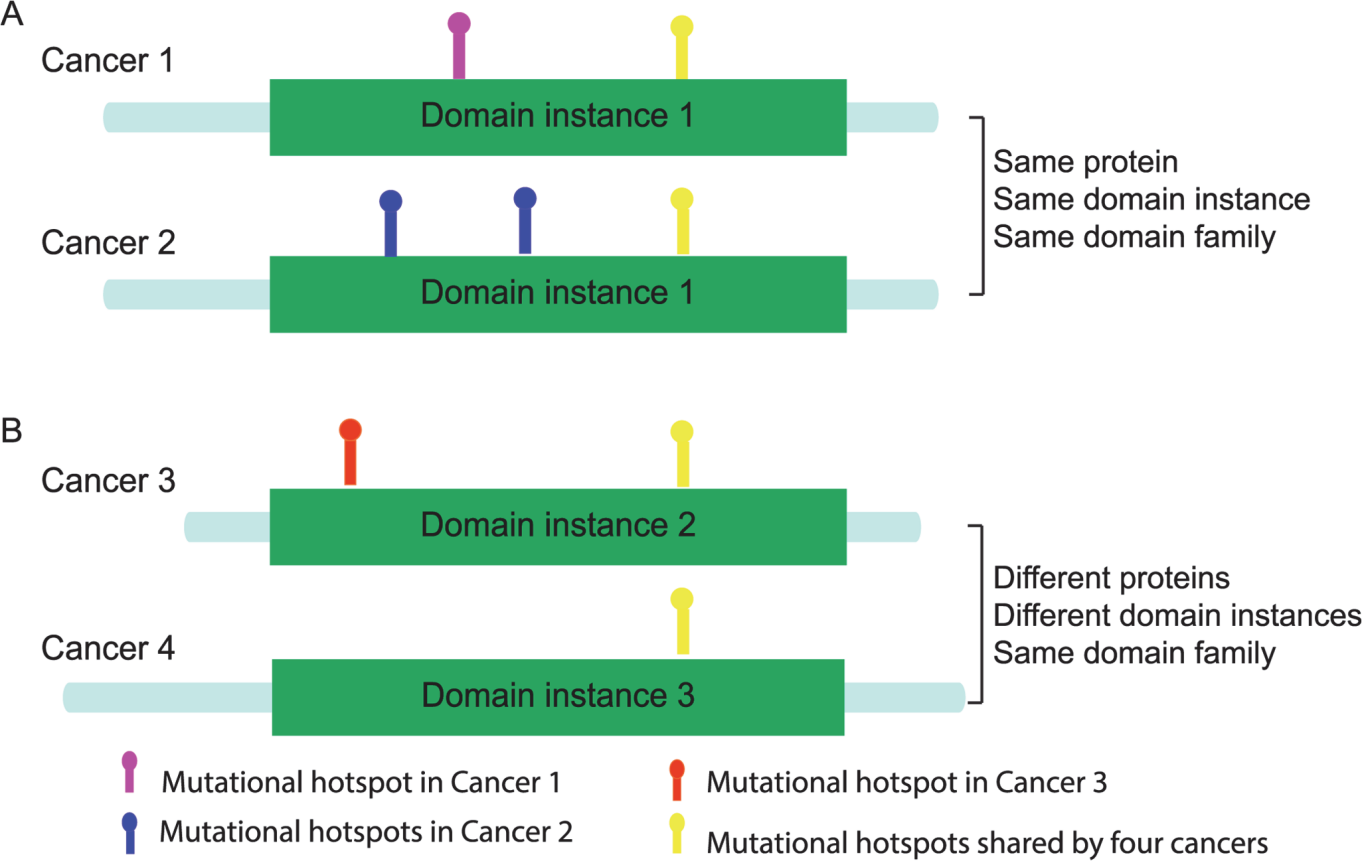
Cancer-type-specific mutational hotspots and mutational hotspots shared by several cancer types. A. shows the distribution of mutational hotspots for different cancer types within a given domain instance. B. shows mutational hotspot distribution patterns of different domain instances (encoded by different genes) that each correspond to the same protein domain type. Mutational hotspots are shown as balls and sticks, domain instances are shown as boxes. Mutational hotspots in different colors represent mutations in different cancer types.

We further analyzed genes with at least one cancer-type-specific SMD. More specifically, we identified a collection of 337 cancer-type-specific mutation hotspots in 68 genes, including some hotspots that appeared in several different types of cancer (Fig. 5., S4 Table). For example, in the EGFR protein, residue p.A289 is a mutational hotspot in central nervous system cancer, p.C231 is a mutational hotspot in prostate cancer (Fig. 5.). Both residues fall in the Furin-like domain of the extra-cellular part of EGFR, but at different domain-domain interaction interfaces.

**Fig 6:**
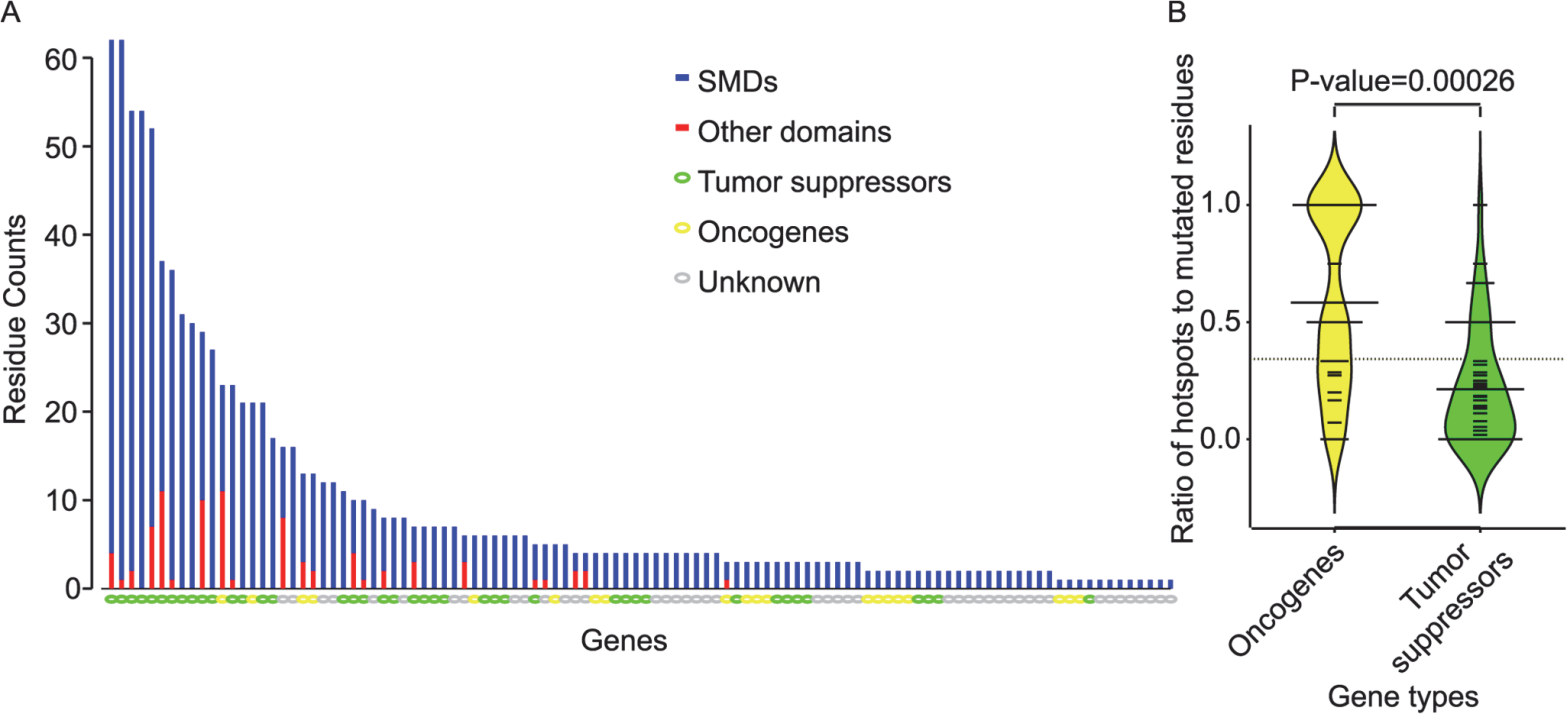
Distribution of mutated residues within a single gene. (A) compares the prevalence with which mutations from a specific cancer type fall within significantly mutated domain instances (SMDs) to the prevalence of mutations in other domain instances. Genes with at least one SMD are represented on *x*-axis in descending order by the number of mutated residues. The length of each blue bar shows the number of the mutated residues falling in SMDs for each cancer type, the length of red bars shown the number of mutated residues falling in other domain instances within the same gene. (B) compares the fraction of mutated residues in SMDs that are hotspots in oncogenes (yellow) and tumor suppressors (green).

It has been proposed that oncoproteins tend to be recurrently mutated at the same amino acid residues, while tumor suppressor proteins tend to be mutated throughout their length[15]. Therefore we systematically compared the mutation pattern between tumor suppressor proteins and oncoproteins. In both tumor suppressor proteins and oncoproteins, mutations were enriched in the SMDs, as expected (Fig. 6A., S5 Table). For each cancer-type-specific SMD, to assess whether mutations were recurrent at a few locations as opposed to being evenly spread, we compared the ratio of mutational hotspots to the total number of mutated residues within domains. We found this ratio to be significantly higher for oncoproteins than for tumor suppressor proteins (Fig. 6B.; *P* = 0.00026, Mann-Whitney U-test). This is consistent with the known tendency of tumor suppressor proteins to carry loss-of-function mutations that can occur in many places, and that of oncoproteins to harbor more specific gain-of-function mutations[15].

**Fig 7:**
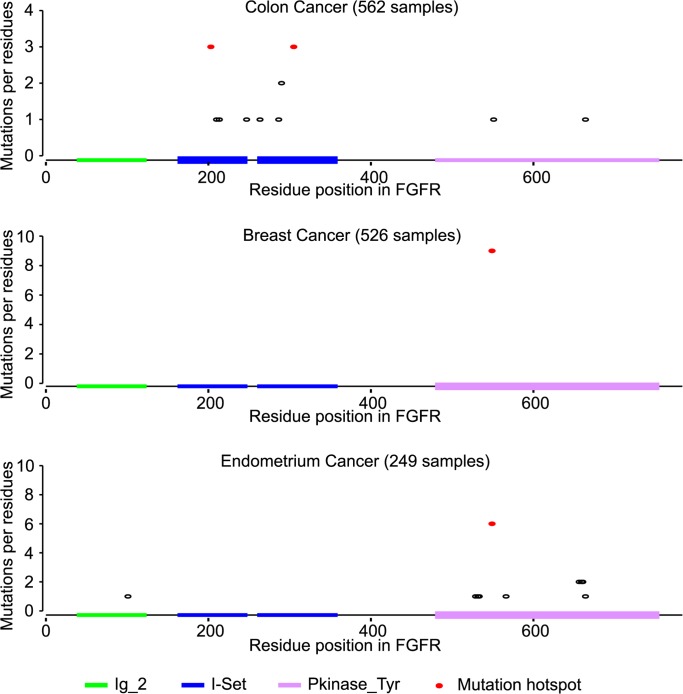
Distribution of mutated residues in FGFR. Sequence positions and frequencies of mutated residues in the FGFR protein are shown. Mutational hotspots for each cancer type are displayed as red dots. SMDs are shown as thicker boxes.

For example, the fibroblast growth factor receptor 2 (FGFR2) is generally regarded as an oncoprotein in breast cancer[15]. Consistent with this view, we found a single hotspot (p.N549) for FGFR2 in breast cancer in the kinase domain, which had not been reported as a hotspot for breast cancer. A previous study of endometrial cancer[54] suggested FGFR2 to be a tumor suppressor protein. Supporting this view, we observed nine evenly-distributed mutated residues in the kinase domain in endometrial cancer, although we also confirm previous observation [54] of the p.N549 hotspot which is more suggestive of an oncoprotein. Four mutational hotspots in the Immunoglobulin I-set domain of FGFR2 were observed in colon cancer, which hints at a tumor suppression role for *FGFR2* in colon cancer (Fig. 7).

We also analyzed the functional properties of the mutational hotspots we observed. Out of the 68 proteins that have at least one mutational hotspot in at least one cancer, we selected 13 proteins for which structures and functional site annotations were available. Of these 13, seven proteins are encoded by oncogenes (*AKT1, BRAF, EGFR, HRAS, KRAS, NRAS*, and *PIK3CA)* and six are encoded by tumor suppressors (*CDKN2A, FBXW7, PTEN, SMAD4, TP53*, and *VHL*). We mapped all observed mutations to protein structures. For tumor suppressor proteins, we found that most mutational hotspots fell at the interface of domain-domain interactions. We also found that, of 47 mutational hotspots, only 3 (6%) fell at functional sites of tumor suppressor proteins (Table 5). For oncoproteins, of 40 mutational hotspots, 15 (38%) fell at functional sites, including GTP/ATP binding sites and other active sites of enzymes. Functional sites were significantly overrepresented among oncogenic mutational hotspots (Odds ratio = 10.0, *P* = 0.0006, Fisher’s Exact Test).

**Table 5:** Genes that encode more than one cancer-type-specific significantly mutated domain instance.

Gene Symbol	Domain Instances per Gene	Significantly Mutated Domain Instance	Primary Site
***TP53***	2	P53	Liver
***TP53***	2	P53_tetramer	Liver
***TP53***	1	P53	Breast
***TP53***	1	P53	Central nervous system
***TP53***	1	P53	**Hematopoietic and lymph**
***TP53***	1	P53	Lung
***TP53***	1	P53	Esophagus
***TP53***	1	P53	Ovary
***TP53***	1	P53	Upper aero-digestive tract
***EGFR***	2	Furin-like	Central nervous system
***EGFR***	2	GF_recep_IV	Central nervous system
***EGFR***	1	Pkinase_Tyr	Lung
***KRAS***	1	Ras	Lung
***KRAS***	1	Ras	Pancreas
***MLL3***	1	zf-HC5HC2H	Breast
***MLL3***	1	zf-HC5HC2H	Prostate
***SMAD4***	1	MH2	Colon
***SMAD4***	1	MH2	Esophagus
***SMARCA4***	1	SNF2_N	Central nervous system
***SMARCA4***	1	SNF2_N	Esophagus
***BCL2***	2	BH4	**Hematopoietic and lymph**
***BCL2***	2	Bcl-2	**Hematopoietic and lymph**
***DDX3X***	2	DEAD	Central nervous system
***DDX3X***	2	Helicase_C	Central nervous system
***PIK3CA***	2	PI3Ka	Endometrium
***PIK3CA***	2	PI3K_p85B	Endometrium

**Fig 8:**
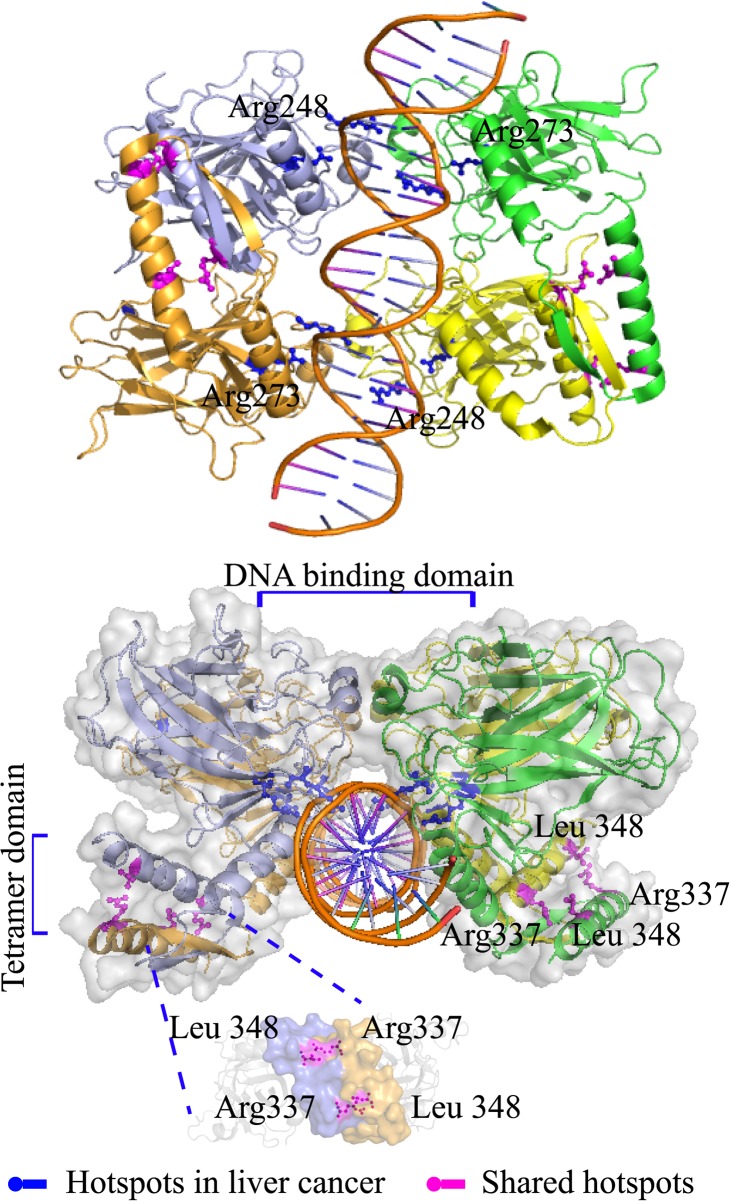
Structural context of p53 protein (PDB 3q05[59]) mutational hotspots. Mutational hotspots shared by eight cancers are displayed as blue sticks. Liver-cancer-specific mutational hotspots are displayed as magenta sticks. The p53 protein structure is colored according to amino acid chain.

The three mutational hotspots detected at known functional sites of a tumor suppressor protein all fell within the p53 protein. The p.R248 and p.R273 hotspots were within the DNA binding site, and have each been reported as sites of potentially oncogenic mutations in many cancer types, including breast cancer[55]. The hotspot p.R337, found in liver cancer, fell within p53’s tetramerization domain, a site of post-translational modification targeted by Protein Arginine N-Methyl Transferase 5 (PRMT5). Methylation of this residue affects the target protein specificity of p53[56, 57]. As shown in Fig. 8., the contact between p.R337 and p.L348, which is a residue in the P53-Tetramerization domain of another chain, may be necessary for tetramerization of the whole protein. The tetramerization of different domains is reported to be essential for the activity of p53[58]. Disruption of tetramerization could have a dominant-negative loss-of-function effect, or a gain- or change-of-function mutation if the un-tetramerized subunits have additional activities. Thus, our analysis points to residue p.R337 being a novel driver mutation in liver cancer.

The different mutational hotspot distribution patterns between oncoproteins and tumor suppressor proteins were generally consistent with the expected gain- and loss-of-function mechanisms of oncogenes and tumor suppressors, respectively[15]. Mutations at functional sites may increase the activation of oncoproteins, while mutations at the inter-chain interfaces may destabilize the protein and lead to loss of function in a tumor suppressor. These distinct mutation patterns can help classify newly identified cancer-associated genes for which oncogene or tumor suppressor roles are unknown. We categorized the ten novel cancer candidate genes that overlap with the Sleeping Beauty dataset based on similarity to the hotspot distribution patterns that are characteristic of oncogenes and tumor suppressors (Table 4). Among the ten genes, seven (*CNTN4, CTNNA3, EPHA6, FOXO1, MAGI1, PTPRD* and *SMAD4*) were predicted to be tumor suppressors. Using transposon insertion positions, the Sleeping Beauty study [60] had annotated three of these seven genes as loss of function (while not suggesting an annotation for the remaining four). We also reported two potential oncogenes, *USP25* and *GNA13* (Table 4). Finally, we identified one gene, *PCDH11X*, for which the domain mutation patterns suggest an oncogenic role in lymphoma and leukemia but a tumor suppressive role in glioblastoma and medulloblastoma.

### Oncogenic mutational hotspots appearing in multiple cancer types

**Fig 9:**
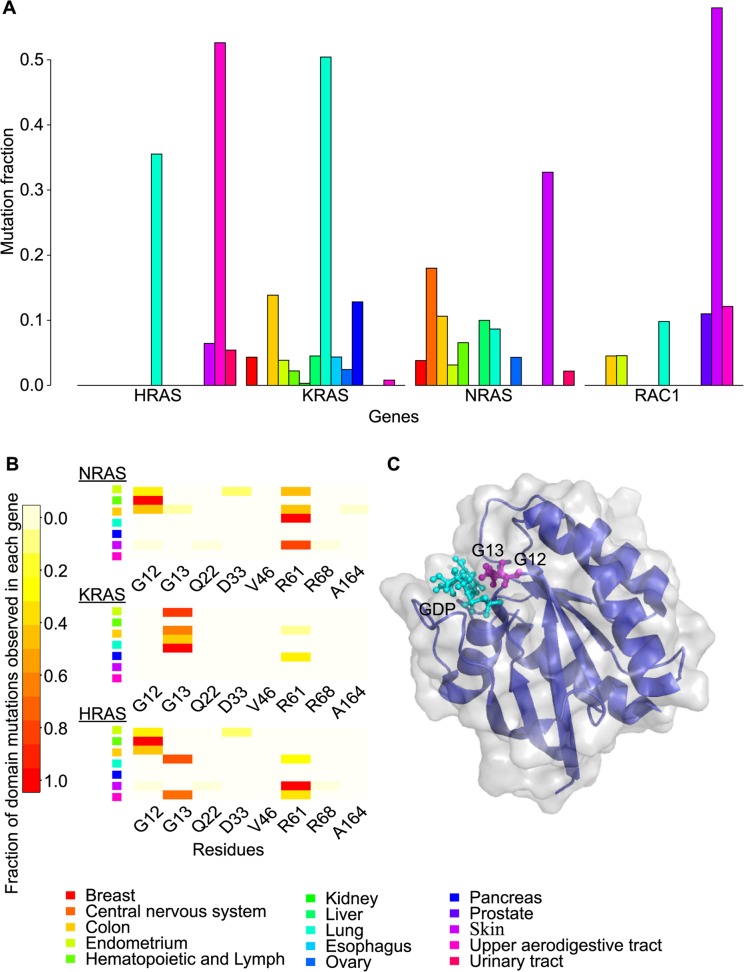
Mutation distributions of different Ras domain instances and the structure of Ras domain. (A) bar graph shows Ras domains encoded by different genes have different mutation rates across cancer types. (B) heat map shows fraction of mutations observed at each residue of a given gene in a given cancer. (C) the structure of the Ras domain encoded by the *KRAS* gene (PDB structural model 4lpk[63]). GTP/GDP binding sites are displayed as magenta sticks, GDP binding sites are colored in cyan.

At the domain level, we noticed that 10 out of 13 oncogenic mutational hotspots shared by at least three cancer types occurred at functional sites (Table 6). This is true not only for domains corresponding to a single gene but also for domain types corresponding to different genes. For example, the Ras domain type (for which instances may be found in multiple genes) was significantly mutated in different cancers (Fig. 9A.). Enrichment of somatic mutations within Ras domains has been reported for different individual genes[61, 62]. Here, we collectively analyzed the domain position-based hotspots for K-RAS, H-RAS, and N-RAS, finding that at least one of the GTP binding site residues p.G12 or p.G13, or the active site residue p.R61 show a relatively high mutation rate in at least five cancer types (Fig. 9B and C.). While each of these three hotspots was known previously for individual genes in individual cancer types, this analysis suggests that an increase in statistical power can be gained in the future by grouping protein domain instances of the same domain type.

**Table 6:** Mutational hotspots observed at functional sites.

Gene Symbol	Category	Mutational Hotspot	Functions
***AKT1***	Oncogene	p.E17	PH–KD interaction
***BRAF***	Oncogene	p.G469	ATP binding
***BRAF***	Oncogene	p.G466	ATP binding
***BRAF***	Oncogene	p.N581	Enzyme-active
***EGFR***	Oncogene	p.G719	ATP binding
***EGFR***	Oncogene	p.G724	ATP binding
***HRAS***	Oncogene	p.G13	GTP binding
***HRAS***	Oncogene	p.Q61	Enzyme-active
***KRAS***	Oncogene	p.G12	GTP binding
***KRAS***	Oncogene	p.G13	GTP binding
***NRAS***	Oncogene	p.G12	GTP binding
***NRAS***	Oncogene	p.Q61	Enzyme-active
***PIK3CA***	Oncogene	p.E545	Intra-molecular binding
***PIK3CA***	Oncogene	p.E542	Intra-molecular binding
***PIK3CA***	Oncogene	p.Q546	Intra-molecular binding
***TP53***	Tumor suppressor	p.R248	DNA binding
***TP53***	Tumor suppressor	p.R273	DNA binding
***TP53***	Tumor suppressor	p.R337	Post-translational modification

Beyond the 10 out of 13 oncogenic mutational hotspots occurring at functional sites, there were three oncogenic mutational hotspots shared by at least three cancer types. They are V600 in Serine/threonine-protein kinase B-Raf (encoded by *BRAF*), and R88 and C420 in the phosphatidylinositol-4,5-bisphosphate 3-kinase encoded by *PIK3CA*. We found both C420 and R88 to be positions of mutational hotspots in endometrium, colon and breast cancer. Although the two residues fall within different domains (C420 in C2 domain, and R88 in p85α domain), they both play important roles in maintaining the p110α/ p85α-iSH2 structure [64], and both are at the binding interface of the C2 and p85α domains. Although each of these mutations has been previously studied as a potential driver mutation in each of these three cancer types, this analysis objectively confirms the ‘hotspot’ status of these mutations.

## Discussion

Major bottlenecks in the systematic study of cancer genomes exist following identification of somatic tumor mutations, including the identification of driver mutations and their functional impacts. By taking advantage of large-scale whole-genome or whole-exome sequencing data and accumulated information about protein structures, we were able to derive and compare the mutational landscapes for 21 cancer types at the domain level. We used a significance test that not only required a domain to have enriched mutational density in a given tumor type relative to other regions in that tumor type, but further required that an enrichment be significantly greater than that observed for all other cancer types taken together. Because region-dependence of mutation rates is similar across tumor types[25], this approach not only identifies cancer-type specific mutational positioning but also implicitly controls for regional differences in mutation rate across the genome.

We analyzed domain types that are significantly mutated in different cancers, such as the Ras domain type and the Pkinase domain type. We found hotspots that were shared between different domain instances in the same domain type, and which appeared in multiple cancer types. By combining this information with protein structure information, we found that all (10 out of 10) such identified hotspots, where they fell within known oncoproteins, are ‘functional hotspots’ in the sense that all fell within ligand-binding or active sites. We also found that, in a given cancer type, a functional hotspot corresponding to a given domain type was never mutated in more than one of the domain instances corresponding to that domain type in the same tumor sample. (Sample information is shown in S1 Table.) This suggests that functional hotpots falling within different genes corresponding to a given domain type may contribute to cancer development by a similar and parallel mechanism, and further suggests that only one mutated functional site might be able to increase the activity of those proto-oncogenes and ultimately contribute to cancer initiation. Functional hotspots included oncogenic mutations within proteins that are generally considered to be tumor suppressors, for example p.R248 and p.R273 in *TP53*. Except for the DNA binding sites p.R248 and p.R273 in the p53 DNA binding domain, we did not find mutational hotspots in known tumor suppressors that appeared in more than five cancer types. Providing greater nuance to previous reports that mutations tend to span the entire tumor suppressor gene[15], we found that tumor suppressor mutations detected in a given cancer type tended to be distributed throughout the entirety of a significantly mutated domain instance, and many mutations occurred within core regions important for the stabilization of the protein complex. Mutations detected in different cancers tended to be focused within domain instances, but were distributed across different domain instances of the same gene product.

Mutational positioning information could assist drug design aimed at precisely targeting the region of the protein involved in a particular cancer. In contrast with gene-level studies of mutational frequency, the domain-level view points to particular functional regions, and identifies tendencies of a gene to be mutated in different regions in different cancers. For most genes, only one domain was found to be significantly mutated in a given cancer type. However, we found five genes that each contain two interaction-mediating domain instances that were significantly mutated in the same cancer type. These five interaction-mediating domain pairs are Bcl-2 and BH4 domains encoded by *BCL2*, which play important roles in regulating cell death and survival[65]; DEAD and Helicase_C domains encoded by *DDX3X*, which play important roles in metabolic processes involving RNAs[66]; and PI3Ka and PI3K_p85B domains encoded by *PIK3CA*, which interact with each other to initiate a vast array of signaling events[67]; Furin-like and GF_recep_IV domains encoded by *EGFR*, which are both extracellular domains of receptor tyrosine protein kinases and which interact with each other to regulate the binding of ligands to the receptor[68]; and finally the DNA binding and P53_tetramer (tetramerization) domains encoded by *TP53*. We also identified 117 domain instance pairs that corresponded to interacting proteins[69], for which at least one member of the pair was an SMD. For most interaction-mediating pairs, only one domain instance was significantly mutated (S6 Table). There are only ten cases where both domains of a predicted interaction-mediating domain pair were significantly mutated in the same cancer type (S7 Table). This result raises the possibility that mutations in those domain instances act by disrupting domain-domain interactions. Distinctive mutation landscapes in different cancers could indicate that tumor development mechanisms across different cancer types are dissimilar, although it is also possible that differences in the mutational spectrum between different cancer types alter the probability of mutation in one domain relative to another.

This domain-level study identified known and novel candidate driver mutations and provided clues to the functional effects of tumor-associated somatic mutations. In total, 41 out of the 100 SMDs we identified are encoded by Cancer Census genes (S1 Table). Among the remaining 59 novel candidate driver genes, many domain instances belong to well-known cancer-associated domain types, such as the Pkinase domain type and the WD40 domain type, supporting the idea that this set contains many cancer driver genes that are not yet annotated as such. By comparing the domain-level mutational landscapes of different cancers generated by our study to previously reported gene-level mutation landscapes in small cell lung cancer, melanoma, colon cancer, and breast cancer[14, 70–73], we noticed at least ten cancer-type-specific SMDs that do not correspond to any previously reported highly mutated cancer-associated genes. For each cancer type, we found at least one new potential cancer-associated domain instance, for example, the diacylglycerol kinase domain encoded by *DGKZ* in chondrosarcoma. The DGKZ protein (using the diacylglycerol kinase domain) usually acts as a sentinel and can control p53 function both during normal homeostasis and during stress response [74]. Other examples include the two cadherin domain instances encoded by *PCDH11X* and *PCDH11Y* in glioblastoma. These domain instances are thought to play important roles in cell-cell communication and are essential for a normally-functioning central nervous system [75]. Also, all the eight tumor samples that contained mutations in *PCDH11Y* (on the Y chromosome) were also mutated at a corresponding position in the X-chromosome homolog *PCDH11X*. Another four (female) samples had mutations detected on both alleles of *PCDH11X* [76]. These alleles each contained one of the novel hotspot mutations p.T486 or p.G442 in the cadherin domain, suggesting the potential role for these hotspot mutations as important recessive driver mutations in glioblastoma.

Because Nehrt *et al*.[32] had previously identified significantly mutated domain types for breast and colon cancer, we wished to assess the novelty of the SMDs we found for these cancer types. Of the 23 SMDs we identified for colon cancer, 20 were novel relative to domain types previously identified by Nehrt *et al* (we confirmed three domain types: the PI3K_p85B domain encoded by *PIK3CA*, the MH2 domain encoded by *SMAD4* and the P53 DNA binding domain encoded by *TP53*). Of the 12 SMDs we identified for breast cancer, only three correspond to a certain highly mutated domain type reported in the study by Nehrt *et al* (the PI3K_p85B domain and PI3Ka domain encoded by *PI3KCA*, and the P53 DNA binding domain encoded by *TP53*). We note that, even where an SMD corresponds to a domain type previously found to be significantly mutated, our analysis in this case identifies individual domain instances as significantly mutated, as opposed to domain types for which the mutations may be spread across multiple genes. In summary, 20 out of 23 (87%) of the colon-cancer associated SMDs, and 9 out of 12 (75%) of the breast-cancer-associated SMDs found here are novel relative to Nehrt *et al*.

Our study also differs from Nehrt *et al*.in that we only reported domain instances for which enrichment relative to other regions was significantly greater in one cancer type than in all other cancer types. This procedure controlled both for mutation rates within each cancer type, and for different rates of mutation across cancer types in each domain relative to others. Although a previous study[25] has pointed to the dangers of candidate driver gene identification through mutation frequency analysis, we note that none of the SMDs we identified fell within the 18 genes for which mutational enrichment was reported to be spurious[25]. In addition to correspondence of the discovered SMDs to known cancer-relevant domain families, our set of novel driver gene candidates overlapped significantly with a large-scale screen for cancer genes based on transposon mutagenesis in mouse. Together, these results indicate that we may be far from having a complete catalogue of cancer-associated genes and that domain-level mutation landscape analysis offers an opportunity to identify new driver genes.

We note that the cancer missense somatic mutation data we mined came from 71 unbiased studies, and that data from unbiased studies tends to contain a higher proportion of passenger mutations compared to data from targeted studies[77]. We therefore chose a relatively conservative significance threshold, necessarily causing us to overlook many candidate driver genes, which might be recovered in the future through larger data sets and use of prior information about cancer relatedness.

## Materials and Methods

To perform the study we first assembled a dataset of somatic mutations. Then, from this dataset we derived a dataset of potentially damaging missense somatic mutations. We analyzed cancer-type-specific SMDs and cancer-type-specific significantly-mutated position-based mutational hotspots. Finally, we analyzed the structural properties of those mutational hotspots.

### Creating the cancer missense somatic mutation dataset

We assembled a total of 237,716 missense somatic mutations in 21 cancer types (Table 7) from 71 whole-genome (WGS) or whole-exome sequencing (WES) studies[8, 14, 16, 24, 70–73, 78–132] included in the COSMIC (Catalogue of Somatic Mutations in Cancer) database (version 67)[127–129]. Most of those studies were conducted by either the International Cancer Genome Consortium (ICGC) [93] or The Cancer Genome Atlas (TCGA) project[136]. The mutations fell within a total of 18,682 genes, corresponding to 22,367 different protein isoforms. Amino acid sequences corresponding to the mutated protein isoforms were also available from the COSMIC database. We used all the protein sequences corresponding to those cancer-associated genes to search against Pfam domain types from the Pfam protein domain family database (version 27) [137], using an *E*-value cutoff of 0.001[138]. A total of 11,633 unique Pfam domain types, encoded by 18,682 mutated genes, were obtained from the Pfam database, considering all transcripts of these genes. Then we mapped the missense somatic mutations to protein domain positions after multiple sequence alignments using HMMER(v3.1b1)[139]. Where a given mutation could be assigned to multiple overlapping domain instances, we mapped the mutation to all of them. Significance of enrichment was calculated separately for each domain instance, so that the results for any given domain instance did not depend on the presence of other overlapping domain instances. We note that the vast majority of all mutations mapped only to a single domain instance (only 6 mutations can be mapped to different domain instances). Finally, among the 11,633 protein domain types, we found 6950 unique Pfam domain types that have at least one missense somatic mutation detected in the studies, all of which had an E-value <0 .0001 (10-fold more stringent than the Pfam-recommended threshold). These 6950 Pfam domain types corresponded to 29,302 unique domain instances. All source code used for extracting missense somatic mutations from COSMIC and mapping them to Pfam domains is provided as supporting material (S1 Protocol).

**Table 7:** Domain position-based mutational hotspots shared by at least three cancers with functional annotations.

Domain Types	Corresponding Genes	Cancer Types	Mutational Hotspot	Function
**Ras domain**	*KRAS, NRAS, HRAS*	8	p.G12	GTP binding
**Ras domain**	*KRAS, NRAS, HRAS*	5	p.G13	GTP binding
**Ras domain**	*KRAS, NRAS, HRAS*	5	p.Q61	Enzyme-active
**PH domain**	*AKT1*	5	p.E17	Intra-molecular binding
**Phosphoinositide 3-kinase family**	*PIK3CA*	7	p.E545	Intra-molecular binding
**Phosphoinositide 3-kinase family**	*PIK3CA*	5	p.E542	Intra-molecular binding
**P53 DNA binding domain**	*TP53*	14	p.R248	Contact with DNA
**P53 DNA binding domain**	*TP53*	9	p.G245	Contact with DNA
**P53 DNA binding domain**	*TP53*	6	p.R273	Contact with DNA
**P53 DNA binding domain**	*TP53*	6	p.R282	Contact with DNA

### Creating the dataset of potentially damaging missense somatic mutations

To predict potential damaging mutations, we used the IntOGen–mutation platform [36, 140], which classified the 237,716 missense somatic mutations into five categories: high, medium, low, unknown and none. We excluded mutations predicted to have no or unknown functional effects from further analyses. This left only 76,158 mutations as potential driver mutations. Those mutations were distributed in 4,509 unique domain types, corresponding to 14,083 genes (Table 1).

### Cancer-type-specific significantly-mutated domain instance analyses

To avoid the possible bias caused by different domain instance lengths and imbalanced sequencing frequency across cancer types, we calculated the cancer-type-specific mutation density as the total number of somatic missense mutations falling in the domain-encoding region of each gene, normalized by the corresponding cumulative domain instance length. We used the Fisher's Exact test to determine whether a certain domain instance is significantly mutated in a given cancer, using the “*stats*” package in R (http://www.r-project.org, [141]). The mutation counts for the R function corresponded to a 2 × 2 contingency table based on whether or not the mutations detected from each cancer type fell (or did not fall) within a given domain instance. We chose a *P*-value threshold (α = 10^–7^) yielding a false discovery rate (FDR) of less than 0.05. We made a heat map representation of the hierarchical clustering of SMDs in different cancers using the “*heatmap.2*” R package based on the −log (*P*-value) of each cancer-type-specific domain instance. We analyzed the tendency of SMDs to co-occur in the same patient sample using Fisher’s Exact test (“*stats*” package in R). Also, genes containing at one or more SMDs were regarded as candidate cancer genes in this study. Overlap between our candidate gene set and Cancer Census genes and the Sleeping Beauty gene sets was also analyzed using the Fisher’s Exact Test (“*stats*” package in R).

### Cancer-type-specific significantly-mutated position based mutational hotspot analyses

We calculated the mutational hotspots within each domain instance encoded by a single gene based on Fisher’s Exact test with a *P*-value cutoff 0.01 (FDR <0.05). False discovery rate analysis was performed using Benjamini & Hochberg FDR[142]. We used the Mann–Whitney U-test to evaluate the significance of difference in distribution patterns of mutation residues between oncoproteins and tumor suppressor proteins. All of these analyses were conducted using the “*stats*” package in R.

### Structural properties for position based mutational hotspots analyses

We downloaded known protein-structure files from the Protein Data Bank[143]. For proteins that had more than one structure file, we chose one, favoring those with larger sequence length and higher crystallographic resolution. For domain-domain interface analysis, mutational hotspots were first mapped onto the available structures by using the Pymol Software (http://www.pymol.org; [144]). The interfacial residues of different domains in different chains were analyzed using Mechismo (http://mechismo.russelllab.org/), ProtInDB (PROTein-protein INterface residues Data Base; Rafael A Jordan, Feihong Wu, Drena Dobbs and Vasant Honavar. unpublished results), and PDBePISA (Proteins, Interfaces, Surfaces and Assemblies; [145]) servers. We retrieved the functional-site information for those mutational hotspots from the Catalytic Site Atlas[146] and the PhosphoSite[147] databases. We used the odds ratio and Fisher’s Exact Test to calculate the tendency of mutational hotspots in oncoproteins to occur at ATP/GTP binding sites or enzyme-active sites, as compared with mutational hotspots in tumor suppressor proteins.

## Supporting Information

S1 Table Mutations that are predicted to compromise the function of the harboring protein. (XLSX)

S2 Table Cancer-type-specific significantly mutated domain instances and corresponding genes in different cancers. For each cancer type this table lists the significantly mutated domain instances (SMDs], corresponding gene symbols, and number of mutations in each domain instance.
(XLSX)

S3 Table Number of cancer types in which each domain instance was significantly mutated. (XLSX)

S4 Table List of mutational hotspots in different cancer types. (XLSX)

S5 Table Number of mutational hotspots and number of mutated residues in each domain instance for each cancer type. (XLSX)

S6 Table List of pairs of significantly mutated domain instances that corresponded to directly-interacting proteins. (XLSX)

S7 Table Ten pairs of domain instances that are inferred to mediate protein interaction with each other, for which both domains in the pair were found to be significantly mutated in the same cancer type. (XLSX)

S1 Protocol A ‘zipped’ file containing source code used for extracting missense somatic mutation information from the COSMIC database, and for mapping mutations to protein domain instances defined by PFAM. (ZIP)
